# Within- and between-twin comparisons of risk for childhood behavioral difficulties after preterm birth

**DOI:** 10.1038/s41390-023-02579-1

**Published:** 2023-04-11

**Authors:** Grace C. Fitzallen, H. Gerry Taylor, Helen G. Liley, Samudragupta Bora

**Affiliations:** 1 School of Psychological Sciences, College of Health and Medicine, University of Tasmania, Launceston, TAS Australia; 2 School of Psychology, Faculty of Health and Behavioural Sciences, The University of Queensland, Brisbane, QLD Australia; 3 Mater Research Institute, Faculty of Medicine, The University of Queensland, Brisbane, QLD Australia; 4 Center for Biobehavioral Health, Abigail Wexner Research Institute at Nationwide Children’s Hospital, and Department of Pediatrics, The Ohio State University, Columbus, OH USA; 5 Department of Pediatrics, University Hospitals Rainbow Babies & Children’s Hospital, Case Western Reserve University School of Medicine, Cleveland, OH USA

## Abstract

**Background:**

Preterm birth and multiple gestation are independently associated with adverse neurodevelopmental outcomes. The objective of this study was to describe risks of screening positive for attention-deficit/hyperactivity disorder (ADHD), autism spectrum disorder (ASD), and anxiety in preterm-born twin children by zygosity (monozygotic, dizygotic) and birth order (first-born, second-born).

**Methods:**

Caregivers of 349 preterm-born twin pairs (42% monozygotic) aged 3–18 years reported child behavioral outcomes on Strengths and Weaknesses of ADHD Symptoms and Normal Behavior; Social Responsiveness Scale, Second Edition; and Preschool Anxiety Scale or Screen for Child Anxiety and Related Emotional Disorders.

**Results:**

Concordance for behavioral outcomes in twin pairs ranged from 80.06 to 89.31% for ADHD, 61.01 to 84.23% for ASD, and 64.76 to 73.35% for anxiety. Monozygotic twins had a greater risk than dizygotic of screening positive for inattention (risk ratio = 2.91, 95% CI = 1.48–5.72) and social anxiety (1.79, 1.23–2.61). Relative to first-born, second-born twins had a greater risk of screening positive for hyperactivity/impulsivity (1.51, 1.06–2.16); overall ASD (2.38, 1.62–3.49); difficulties with social awareness (2.68, 1.94–3.71), social cognition (4.45, 3.06–6.46), and social communication (2.36, 1.56–3.57); restricted/repetitive behavior (1.91, 1.30–2.81); overall anxiety (1.34, 1.10–1.64); generalized anxiety (1.34, 1.11–1.60); and social anxiety (1.32, 1.06–1.64).

**Conclusion:**

The current findings emphasize considering zygosity and birth order in preterm and multiple birth outcomes research, and highlight clinical implications for discharge planning, neurodevelopmental surveillance, and facilitating parenting and family support.

**Impact:**

- Zygosity and birth order are important determinants of behavioral and socioemotional outcomes in preterm-born twins.
- Among 349 preterm-born twin pairs aged 3–18 years (42% monozygotic), 61–89% demonstrated concordance for behavioral and socioemotional outcomes.
- Monozygosity had greater risks than dizygosity for positive screening of inattention and social anxiety.
- Second-born twins had greater risks than first-born for hyperactivity/impulsivity, social difficulties (awareness, cognition, communication), restricted/repetitive behavior, and anxiety (generalized, social).
- These findings have implications for discharge planning, neurodevelopmental surveillance, and facilitating parenting and family support.

## Introduction

Preterm birth (<37 weeks gestation) is associated with an elevated risk for a range of behavioral and socioemotional difficulties from childhood through to young adulthood, compared with healthy term birth.^1^ This risk is greatest for attention-deficit/hyperactivity disorder (ADHD), autism spectrum disorder (ASD), and anxiety disorders.^2^ Recent meta-analyses show that children born preterm are two-to-four times more likely than term-born peers to meet full diagnostic criteria for these conditions, with risk increasing with lower gestational age.^1,3–6^

Worldwide, the number of survivors after preterm birth continues to rise,^7,8^ attributable to improvements in perinatal and neonatal care. This has resulted in increased survival of infants born extremely preterm at progressively earlier weeks of gestation.^9–11^ Furthermore, in some countries, there has been substantial growth in the range, accessibility, and use of assisted reproductive technologies, in addition to increasing maternal age at childbearing, both of which are associated with higher rates of preterm birth and multiple gestation.^12–16^ Increased uptake of reproductive technologies has seen a two-fold increase in the incidence of multiple birth,^17^ which now accounts for approximately 20% of all preterm births.^8^

Multiple gestation pregnancies have implications for economic and parenting outcomes. For example, the cost associated with multiple birth is almost three times that for singleton birth over the first postnatal year, with further costs incurred for the use of reproductive technologies.^18–22^ Furthermore, it is established that preterm birth is associated with increased risk for maternal psychopathology^23,24^ and maladaptive parenting behavior,^25^ which are known risk factors for suboptimal child development.^26,27^ Research shows that these adverse experiences are exacerbated for mothers who deliver multiple birth neonates preterm, compared with singleton neonates preterm.^28^ Specifically, parents of preterm-born twins exhibit increased parenting stress,^29–31^ reduced responsiveness to the child, and disrupted mother-infant interactions,^29,32^ compared with parents of preterm-born singletons. These disruptions have been shown to impact the development of children at 18 months of age.^29,32^ Nevertheless, when evaluating early child neurodevelopment, few or no significant differences in sequelae have been found between preterm-born multiples and singletons.^33–35^

While there are many antenatal and intrapartum factors impacting twin gestations, zygosity and birth order are the two core factors for consideration when examining childhood sequelae. Zygosity is used to evaluate heritability, commonly determined by the concordance of outcomes. Concordance is typically greater for monozygotic twins (derived from a single zygote) than for dizygotic twins (derived from two separate zygotes).^36–38^ Birth order, defined as the order in which twins are birthed to the extra-uterine environment (nonsynonymous to in utero labeling of Twin A and Twin B), has also been shown to contribute to neonatal outcomes that may have long-term sequelae. Specifically, research consistently demonstrates that second-born infants within a twin pair face significantly greater risk than first-born for infant mortality^39^ and neonatal clinical risk (i.e., anoxia, fetal distress, lower birthweight, lower Apgar scores).^40–42^

To date, most studies evaluating the risk for ADHD, ASD, and anxiety in children born preterm have not examined the role of heritability or multiple birth factors. In the healthy typical population, twin research of zygosity suggests an established rate of heritability for these outcomes ranging between 50 and 90%.^43–47^ While rates for these outcomes are significantly elevated for children born preterm, there has been a paucity of research investigating the role of preterm birth in conjunction with multiple gestation. This is further confirmed by recent systematic reviews and meta-analyses in their limitation to synthesize the risks associated with multiple gestation in the prevalence of ADHD, ASD, and anxiety in preterm-born children and adolescents.^1,3–6,48^

Therefore, to facilitate research on the intersection between preterm birth and multiple gestation, we investigate behavioral and socioemotional outcomes of preterm-born twin children between 3 and 18 years of age using a cross-sectional study design. Our first aim was to compare the within-twin pair concordance for ADHD, ASD, and anxiety outcomes by zygosity (monozygotic, dizygotic). Of particular interest was to compare the rates of concordance between monozygotic and dizygotic twins of screening positive for symptomatology (i.e., both children within a twin pair classified as at-risk) of each of these outcomes. Our second aim was to examine the role of birth order by comparing rates of screening positive for ADHD, ASD, and anxiety symptomatology between first-born and second-born twins (regardless of zygosity status). It was hypothesized that monozygotic twins would have higher concordance for ADHD, ASD, and anxiety outcomes than dizygotic twins, and second-born twins would have a higher risk than first-born twins of screening positive for symptomatology of each of these outcomes.

## Methods

### Participants

Primary caregivers of children born preterm (<37 weeks gestation) were recruited online through parent organizations between October 2019 and February 2020 to report on their children’s behavioral and socioemotional outcomes. Caregivers had to be between 22 and 63 years of age at assessment, with primary residence in Australia, New Zealand, Canada, the United States of America, the United Kingdom, or Ireland, and English as their primary language (not necessarily native). For this study, participants were eligible if twins in their care were born preterm, aged 3–18 years and both twin children surviving at assessment, with no known history of chromosomal anomaly, fetal alcohol spectrum disorders, and/or intellectual disability (IQ <70). Furthermore, caregiver reports of the child’s gestational age at birth had to be consistent at both screening and outcomes assessment, across two different response formats, along with a response of “confident”, “very confident”, or “extremely confident” on a 5-point Likert scale.

Overall, 627 primary caregivers of 3–18-year-old twins born preterm were invited to participate in this study. The response rate was 62.67% (*n* = 393/627). Of responders, 7.34% (*n* = 24/393) were ineligible (deceased twin, *n* = 16; child IQ <70, *n* = 4; incongruent gestational age reporting, *n* = 4). Of those eligible (*n* = 369), data from 20 caregivers were further excluded because they did not complete all questionnaires for one twin. Therefore, the final sample for this study comprised 698 children born preterm from 349 twin pairs.

We adhered to the American Association for Public Opinion Research’s best practices for survey research to inform the study protocol. All procedures were approved by The University of Queensland Human Research Ethics Committee. All study participants provided informed consent.

### Measures

All data collected for this study, including child neonatal and concurrent characteristics, were reported by primary caregivers. Primary caregiver-reported child behavioral and socioemotional outcomes data were collected at a single time-point on three age-appropriate standardized instruments with strong psychometric properties, including concurrent validity with the Diagnostic and Statistical Manual of Mental Disorders, Fourth Edition and Fifth Edition diagnoses.^49–56^ A secure web-based portal was used to administer these instruments. To minimize response bias, a wash-out period of 2 months was used between reporting on each twin, with all caregivers reporting on their second-born twin first. Caregivers were asked to consider their child’s behavior in the past 6 months. Scores were classified as typically developing or at-risk based on the recommended cut-off criterion for each norm-referenced instrument.

ADHD symptomatology was evaluated using the Strengths and Weaknesses of ADHD Symptoms and Normal Behavior Scale.^56^ This 18-item instrument includes subdomains of inattention and hyperactivity/impulsivity, with screening positive across both subdomains classified as Combined ADHD. Caregivers rated the extent to which each item described their child’s behavior on a 4-point Likert scale from “not at all” to “very much”.

ASD symptomatology was evaluated using the Social Responsiveness Scale, Second Edition.^57^ This 65-item instrument includes subdomains of social awareness, social cognition, social communication, social motivation, and restricted/repetitive behavior. Caregivers rated each item on a 4-point Likert scale from “not true” to “almost always true”.

Anxiety symptomatology was evaluated using two age-appropriate instruments. Caregivers of children aged 3–7 years completed the 34-item Preschool Anxiety Scale,^55^ rating items on a 5-point Likert scale from “not true at all” to “very often true”. The total score summates developmentally appropriate subdomain scores of generalized anxiety, separation anxiety, social anxiety, obsessive-compulsive symptoms, and physical injury fears.

Caregivers of children aged 8–18 years completed the 41-item Screen for Child Anxiety and Related Emotional Disorders,^58^ rating items on a 3-point Likert scale from “not true or hardly ever true” to “very true or often true”. The total score encompasses subdomains of generalized anxiety, separation anxiety, social anxiety, panic and somatic symptoms, and school avoidance. Our analysis was restricted to common subdomains across the two instruments: generalized anxiety, separation anxiety, and social anxiety.

Zygosity status (monozygotic vs. dizygotic) of twin pairs was determined based on the primary caregiver-reported judgment of the child’s zygosity. Responses were validated using the adapted Zygosity Questionnaire for Young Twins.^59^

### Statistical analyses

Data analysis was completed using the IBM^®^ SPSS^®^ version 24.0. Between-group differences in rates for within-twin concordance of outcomes by zygosity (monozygotic, dizygotic) were examined using the *χ*^2^ test of independence with risk ratio (RR) and 95% confidence interval (CI) as the measure of effect size. Concordance was scored if both members of the twin pair were categorized as typically developing or both were categorized as at-risk. Discordance was scored if one member of the twin pair was categorized as typically developing and the other as at-risk or vice versa. Birth order comparisons using McNemar’s test on all paired outcomes were used to determine if first-born or second-born twins were more at risk of screening positive for symptomatology. A *p* < 0.05 was defined as the threshold of statistical significance for all analyses, with no adjustment for multiple comparisons because of the exploratory nature of this study. All analyses were conducted according to a priori hypotheses.

## Results

### Sample characteristics

As shown in Table 1, 41.83% (*n* = 146/349) of twin pairs were monozygotic. Consistent with predictions for sex discordance within twins, there were 36.10% male–male pairs, 22.92% male–female, and 40.97% female–female. Regarding gestational age, 11.75% and 29.23% of twin pairs were born extremely preterm and very preterm, respectively. Table 2 compares sample characteristics by birth order. Among twin pairs, second-born twins were significantly more likely to have a physical or motor disability without cerebral palsy (4.03% vs. 0.68%) and speech-language impairment (22.48% vs. 13.27%) relative to first-born twins.

**Table 1:** Characteristics of the sample.

Characteristics, % (numerator/denominator)	*N* = 349 twin pairs
*Child*	
Gestational age, mean ± SD, weeks	32 ± 3
Extremely preterm, <28 weeks	11.75 (41/349)
Very preterm, 28–31 weeks	29.23 (102/349)
Moderate/late preterm, 32–36 weeks	59.03 (206/349)
Birthweight discordance ≥400 g	22.37 (66/295)
Sex discordance	
Male–male	36.10 (126/349)
Male–female	22.92 (80/349)
Female–female	40.97 (143/349)
Zygosity	
Monozygotic	41.83 (146/349)
Dizygotic	58.17 (206/349)
Chronological age at assessment, mean ± SD, years	7 ± 4
*Maternal at childbirth*	
Maternal age, mean ± SD, years	32 ± 4
Minority race/ethnicity	6.98 (24/344)
Low education [high school graduate or below]	10.89 (38/349)
Low family socioeconomic status [unemployed, unskilled, semi-skilled]	8.60 (30/349)
Single parent family	4.30 (15/349)

**Table 2:** Characteristics of first-born and second-born children within a twin pair.

Characteristics, % (numerator/denominator)	First-born twin [*n* = 349]	Second-born twin [*n* = 349]	*p*
Child neonatal			
Birthweight, mean ± SD, g	1750 ± 584 [*n* = 295]	1620 ± 590 [*n* = 349]	
Male sex	47.56 (166/349)	47.56 (166/349)	1.0
Confirmed neonatal infection	14.75 (41/278)	19.21 (63/328)	0.19
Oxygen therapy at 36 weeks	27.40 (80/292)	32.25 (109/338)	0.30
Severe brain injury or abnormality	3.47 (10/288)	2.88 (10/347)	1.0
Child concurrent			
Blindness	0.68 (2/294)	0.58 (2/347)	1.0
Deafness	0 (0/294)	1.15 (4/347)	–
Cerebral palsy	2.38 (7/294)	2.02 (7/347)	1.0
Physical or motor disability without cerebral palsy	0.68 (2/294)	4.03 (14/347)	0.004
Learning disability	5.44 (16/294)	6.92 (24/347)	0.70
Speech/language impairment	13.27 (39/294)	22.48 (78/347)	<0.001
Intervention for >6 months			
Behavioral counseling	2.04 (6/294)	0.86 (3/347)	0.51
Mental health intervention	3.40 (10/294)	3.75 (13/347)	0.65
Occupational therapy	15.31 (45/294)	17.00 (59/347)	0.27
Physical therapy	10.88 (32/294)	11.82 (41/347)	0.47
Remedial education	2.38 (7/294)	3.75 (13/347)	0.39
Speech and language therapy	20.07 (59/294)	15.85 (55/347)	0.09

### Outcomes by zygosity

As shown in Table 3, there was moderate to high within-twin pair concordance for all outcomes (80.06–89.31% for ADHD, 61.01–84.23% for ASD, and 64.76–73.35% for anxiety). Concordance was significantly greater for monozygotic than dizygotic twins for combined ADHD (93.75% vs. 86.14%), hyperactivity/impulsivity (89.58%, vs. 81.68%), inattention (88.19% vs. 74.26%), restricted/repetitive behavior (89.29% vs. 76.53%), and social anxiety (78.77% vs. 68.47%).

**Table 3:** Concordance and risk for behavioral and socioemotional outcomes within a twin pair by zygosity.

Characteristics, % (numerator/denominator)	Monozygotic twin pair [*n* = 146]	Dizygotic twin pair [*n* = 203]	*p*	Risk ratio (95% confidence interval)	Total sample [*N* = 349 twin pairs]
ADHD					
Combined	93.75 (135/144)	86.14 (174/202)	0.02	1.09 (1.02–1.17)	89.31 (309/346)
Hyperactivity/impulsivity	89.58 (129/144)	81.68 (165/202)	0.04	1.10 (1.01–1.20)	84.97 (294/346)
Inattention	88.19 (127/144)	74.26 (150/202)	0.001	1.19 (1.07–1.31)	80.06 (277/346)
ASD					
Overall	82.01 (114/139)	76.92 (150/195)	0.26	1.07 (0.96–1.19)	79.04 (264/334)
Social awareness	62.86 (88/140)	59.69 (117/196)	0.56	1.05 (0.89–1.25)	61.01 (205/336)
Social cognition	62.86 (88/140)	65.31 (128/196)	0.64	0.96 (0.82–1.13)	64.29 (216/336)
Social communication	86.33 (120/139)	77.95 (152/195)	0.05	1.11 (1.00–1.22)	81.44 (272/334)
Social motivation	88.57 (124/140)	81.12 (159/196)	0.07	1.09 (0.99–1.19)	84.23 (283/336)
Restricted/repetitive behavior	89.29 (125/140)	76.53 (150/196)	0.003	1.17 (1.06–1.28)	81.85 (275/336)
Anxiety					
Overall	73.97 (108/146)	70.94 (144/203)	0.53	1.04 (0.92–1.19)	72.21 (252/349)
Generalized anxiety	69.86 (102/146)	61.08 (124/203)	0.09	1.14 (0.98–1.33)	64.76 (226/349)
Social anxiety	78.77 (115/146)	68.47 (139/203)	0.03	1.15 (1.01–1.30)	72.78 (254/349)
Separation anxiety	76.71 (112/146)	70.94 (144/203)	0.23	1.08 (0.95–1.23)	73.35 (256/349)

When restricting current analyses to within-twin pair concordance of at-risk screening categorization only (i.e., excluding “typically developing” concordant sample; Table 4), monozygotic twins were more likely than dizygotic of screening positive for inattention (RR = 2.91, 95% CI = 1.48–5.72) and social anxiety (RR = 1.79, 95% CI = 1.23–2.61). Furthermore, although monozygotic twins demonstrated relatively higher concordance of screening positive for combined ADHD symptomatology than dizygotic twins, the between-groups difference did not reach statistical significance (*p* = 0.08). Finally, although not statistically significant (*p* = 0.07), social cognition was the only domain where dizygotic twins had a higher concordance of screening positive for difficulties than monozygotic twins (18.07% vs. 7.14%).

**Table 4:** Concordance and risk of screening positive for behavioral and socioemotional symptomatology within a twin pair by zygosity.

Characteristics, % (numerator/denominator)	Monozygotic twin pair	Dizygotic twin pair	*p*	Risk ratio (95% confidence interval)	Total sample
ADHD					
Combined	43.75 (7/16)	20.00 (7/35)	0.08	2.19 (0.92–5.19)	27.45 (14/51)
Hyperactivity/impulsivity	42.31 (11/26)	31.48 (17/54)	0.34	1.34 (0.74–2.44)	35.00 (28/80)
Inattention	46.88 (15/32)	16.13 (10/62)	0.001	2.91 (1.48–5.72)	26.60 (25/94)
ASD					
Overall	21.88 (7/32)	21.05 (12/57)	0.93	1.04 (0.46–2.37)	21.35 (19/89)
Social awareness	7.14 (4/56)	7.06 (6/85)	0.99	1.01 (0.30–3.43)	7.09 (10/141)
Social cognition	7.14 (4/56)	18.07 (15/83)	0.07	0.40 (0.14–1.13)	13.67 (19/139)
Social communication	26.92 (7/26)	17.31 (9/52)	0.32	1.56 (0.65–3.71)	20.51 (16/78)
Social motivation	36.00 (9/25)	17.78 (8/45)	0.09	2.03 (0.89–4.59)	24.29 (17/70)
Restricted/repetitive behavior	37.50 (9/24)	17.86 (10/56)	0.06	2.10 (0.98–4.50)	23.75 (19/80)
Anxiety					
Overall	47.22 (34/72)	42.72 (44/103)	0.56	1.11 (0.79–1.54)	44.57 (78/175)
Generalized anxiety	43.59 (34/78)	37.30 (47/126)	0.37	1.17 (0.83–1.64)	39.71 (81/204)
Social anxiety	54.41 (37/68)	30.43 (28/92)	0.002	1.79 (1.23–2.61)	40.63 (65/160)
Separation anxiety	50.00 (34/68)	46.36 (51/110)	0.64	1.08 (0.79–1.47)	47.75 (85/178)

### Outcomes by birth order

As shown in Table 5, higher rates of screening positive for behavioral and socioemotional difficulties across both domain and subdomain levels were consistently evident for second-born relative to first-born children within a twin pair. At the domain level, second-born twins had a significantly greater risk of screening positive for ASD (RR = 2.38, 95% CI = 1.62–3.49) and anxiety (RR = 1.34, 95% CI = 1.10–1.64). Furthermore, although second-born twins demonstrated an elevated risk of screening positive for combined ADHD compared with first-born twins, between-group differences did not reach statistical significance (*p* = 0.10).

**Table 5:** Rates of screening positive for behavioral and socioemotional symptomatology within a twin pair by birth order.

Characteristics, % (numerator/denominator)	First-born twin [*n* = 349]	Second-born twin [*n* = 349]	*p*	Risk ratio (95% confidence interval)	Total sample [*N* = 349 twin pairs]
ADHD					
Combined	7.80 (27/346)	10.98 (38/346)	0.10	1.41 (0.88–2.25)	9.39 (65/692)
Hyperactivity/impulsivity	12.43 (43/346)	18.79 (65/346)	0.004	1.51 (1.06–2.16)	15.61 (108/692)
Inattention	14.74 (51/346)	19.65 (68/346)	0.05	1.33 (0.96–1.86)	17.20 (119/692)
ASD					
Overall	9.58 (32/334)	22.75 (76/334)	<0.001	2.38 (1.62–3.49)	16.17 (108/668)
Social awareness	12.20 (41/336)	32.74 (110/336)	<0.001	2.68 (1.94–3.71)	22.47 (151/672)
Social cognition	8.63 (29/336)	38.39 (129/336)	<0.001	4.45 (3.06–6.46)	23.51 (158/672)
Social communication	8.38 (28/334)	19.76 (66/334)	<0.001	2.36 (1.56–3.57)	14.07 (94/668)
Social motivation	12.20 (41/336)	13.69 (46/336)	0.58	1.12 (0.76–1.66)	12.95 (87/672)
Restricted/repetitive behavior	10.12 (34/336)	19.35 (65/336)	<0.001	1.91 (1.30–2.81)	14.73 (99/672)
Anxiety					
Overall	30.95 (108/349)	41.55 (145/349)	<0.001	1.34 (1.10–1.64)	36.25 (253/698)
Generalized anxiety	34.96 (122/349)	46.70 (163/349)	<0.001	1.34 (1.11–1.60)	40.83 (285/698)
Social anxiety	27.79 (97/349)	36.68 (128/349)	0.002	1.32 (1.06–1.64)	32.23 (225/698)
Separation anxiety	34.96 (122/349)	40.40 (141/349)	0.06	1.16 (0.95–1.40)	37.68 (263/698)

At the subdomain level, second-born twins had a significantly greater risk than first-born twins of screening positive for hyperactivity/impulsivity (RR = 1.51, 95% CI = 1.06–2.16); difficulties in social awareness (RR = 2.68, 95% CI = 1.94–3.71), social cognition (RR = 4.45, 95% CI = 3.06–6.46), and social communication (RR = 2.36, 95% CI = 1.56–3.57); restricted/repetitive behavior (RR = 1.91, 95% CI = 1.30–2.81); generalized anxiety (RR = 1.34, 95% CI = 1.11–1.60); and social anxiety (95% CI = 1.32, 1.06–1.64). Furthermore, although not statistically significant, second-born twins demonstrated elevated risk of screening positive for inattention (19.65% vs. 14.74%) and separation anxiety (40.40% vs. 34.96%) relative to first-born twins.

## Discussion

In this cross-sectional study of preterm-born twin pairs, we found moderate to high rates of agreement between children within a twin pair for behavioral and socioemotional outcomes. This agreement was greater for monozygotic than dizygotic twins for 12 of the 13 outcomes of interest, with five between-group differences reaching statistical significance. Of note, the agreement was higher for monozygotic than dizygotic twins on all ADHD outcomes (i.e., combined ADHD, hyperactivity/impulsivity, inattention). These findings align with previous twin and adoption research suggesting an enduring genetic influence on ADHD, over and above the influence of shared childhood environmental factors.^60^ Furthermore, exposures related to shared physiological threats, such as those possibly due to placental vascular connections, may have also played a role. Interestingly, when subgroup analyses were performed to evaluate within-twin pair agreement for the at-risk concordant sample alone, the agreement was still greatest for monozygotic twins for inattention but not for hyperactivity/impulsivity. This corresponds to existing literature suggesting that preterm-born children face an elevated risk for the inattentive subdomain of ADHD than the hyperactive/impulsive subdomain.^2^ Furthermore, susceptibility to inattention may be explained by distinct etiologic pathways underlying the two subdomains of ADHD.^45^

A different risk profile was evident for ASD and anxiety symptomatology (in terms of within-twin pair concordance), whereby statistically significant between-group differences for monozygotic and dizygotic twins were evident for only two of the ten outcomes of interest. Our findings are incongruent with previous studies reporting greater rates of heritability for these outcomes than ADHD, and considerable genetic overlap with ADHD.^45^ Previous research supports the strong influence of early environmental factors on the development of social and anxiety difficulties.^61–63^ Increased social engagement opportunities because of having a twinsibling and differential maternal parenting behavior as a result of preterm birth may be possible contributors. For example, 2-year-old preterm-born twins have been found to engage in social play behaviors at a similar level to singletons.^30^ This may indicate that exposure to social play has a protective effect on social development and competence, that overrides any effect of zygosity. Furthermore, preterm birth is associated with different maternal parenting behaviors compared with those exhibited after healthy term birth, including reduced maternal sensitivity and responsiveness^64,65^ and increased hypervigilance of the preterm-born child.^26^ Both of these adverse changes in parenting behaviors are exacerbated for mothers after multiple birth.^28,29,32^ These differential patterns of maternal behaviors can negatively impact a child’s opportunity to learn and model appropriate social behaviors, which may adversely impact social competence and worsen symptomatology associated with ASD. They may also interfere with children’s ability to learn how to manage and regulate their emotions which can manifest as anxiety.^4^ Just as twinsibling interaction may be protective, altered parenting interactions may have a pervasive negative influence on existing genetic predisposition.

Interestingly, we found monozygotic twins were more likely to have a greater risk of screening positive for social anxiety symptomatology but not for any social subdomains of ASD (i.e., social awareness, social cognition, social communication, and social motivation). This discrepancy may be explained by alterations, described in association with preterm birth, in the growth and development of brain areas implicated in the production of anxiety responses, including the amygdala.^66,67^

A possible limitation of this study is our reliance on primary caregiver-reported zygosity. These reports may have, in many cases, been based on typically reliable methods such as ultrasound or placental pathological demonstration of chorionicity. While highly reliable DNA fingerprints, microsatellites, and nucleotide polymorphisms are more accurate, they are very rarely used clinically.^68^ Nevertheless, given the proportion of monozygotic and dizygotic twins reported by caregivers in this study, Hardy-Weinberg principles lead to a prediction of 29% sex-discordant twins, similar to 23% in our sample. This suggests that the attribution of zygosity within this study was largely representative.

Caregiver responses suggest that second-born twins were at greater risk for behavioral and socioemotional difficulties than first-born twins. This may be partly explained by higher rates of concurrent motor and language impairments among second-born twins in our sample. Differential parenting may have also played a role, although twin research suggests an elevated risk for second-born twins for adverse early neurodevelopment as a consequence of biological factors such as birth complications associated with in utero maturation and delivery method.^42,69–71^ Twin studies on ADHD, ASD, and anxiety primarily focus on discordant birthweight as an explanation for differences in outcomes within twin pairs; however, larger birthweight is not associated with first birth order. Due to the cross-sectional nature of this study, we were unable to evaluate antenatal and intrapartum factors that may have moderated the association between second birth order and childhood behavioral outcomes. These factors include fetal growth restriction, placentation, premature rupture of membranes, twin-to-twin transfusion syndrome, and twin anemia polycythemia sequence. Furthermore, while efforts were made to ensure a robust collection of caregiver-reported information, findings on birth order differences must be interpreted with caution and warrant future investigations using more objective methods such as medical records (i.e., placental confirmation), data linkage, or prospective cohort designs.

Despite our novel findings based on a large sample and outcomes assessment using well-validated behavioral screening instruments, methodological limitations need to be acknowledged during interpretation. First, there is the potential for selection bias due to the exclusion of twin pairs with IQ <70. Because there is high co-occurrence and heritability of ASD and intellectual disability,^72,73^ this may have resulted in the exclusion of children with the greatest risk. Furthermore, it is plausible that there may have been recall bias for ADHD-related behaviors, which are typically more noticeable due to their externalizing nature compared with anxiety and ASD, which predominantly manifest in an internalized manner. Another limitation is that differences detected in outcome domains may have occurred because of measurement variance from using different standardized instruments, and not utilizing counterbalancing or randomization of instrument order or twin reference. Finally, the use of caregiver-reported responses on screening instruments rather than comprehensive diagnostic assessment (i.e., psychiatric interview, collateral information by teacher report, etc.) may have resulted in detection bias.

Taken together, our novel findings suggest that twin birth impacts the risk for behavioral and socioemotional difficulties in children after preterm birth, with risks greater in monozygotic than dizygotic and second-born than first-born twins. Higher concordance among monozygotic twins suggests strong genetic influence; nonetheless, there were sufficient differences in concordance across subdomains indicating a major impact of environmental influences. The current findings should be replicated using prospective, large-scale cohort study designs utilizing diagnostic evaluations to better understand the role of genetics and epigenetics, or other environmental factors on the risk for ADHD, ASD, and anxiety after preterm birth. Our findings highlight the importance of considering the role of zygosity and birth order in all neonatal follow-up research, including exercising caution when adopting family-based cluster analysis approaches for examining child-based outcomes. Furthermore, once there is a more comprehensive understanding of these outcomes in preterm-born twin children, there may be clinical implications for neonatal care, discharge planning, neurodevelopmental surveillance, and facilitating parenting and family support. Specifically, with increasing rates of viable multiple births, it is becoming increasingly important for medical and allied health professionals to consider differential planning and monitoring in the growth and development of twin children to optimize long-term outcomes.

## Data Availability

Data corresponding to the current analyses will be available on request from the corresponding author.
